# Halal Perspectives on Grasshoppers (Locusts): A Path to Sustainable and Nutritionally Acceptable Alternative Protein Source

**DOI:** 10.1111/1541-4337.70283

**Published:** 2025-09-09

**Authors:** Mian Nadeem Riaz, Fariha Irshad, Awis Qurni Sazil

**Affiliations:** ^1^ Department of Food Science and Technology Texas A & M University College Station Texas USA; ^2^ Halal Products Research Institute Universiti Putra Malaysia Serdang Malaysia

## Abstract

The food system is under increased pressure because of the need for sustainability, greater food safety, and increasing need for protein sources. Grasshopper‐based food products are becoming a new option. Products made from grasshoppers represent a sustainable and nutritious alternative to traditional livestock. Besides offering added benefits such as reduced water and land use and minimal greenhouse gas emissions, they also have high levels of protein. While locusts are clearly considered halal in Islamic tradition, the halal status of grasshoppers, though they belong to the same biological family, is subject to scholarly interpretation. Locusts are a specific type of grasshopper that can enter a swarming phase; not all grasshoppers are locusts, and this distinction is significant in Islamic dietary law. Locusts, which are a type of grasshopper, are considered halal; therefore, they represent a potentially viable protein source in many countries with large Muslim populations. The halal status of other grasshopper species, however, remains subject to scholarly interpretation.

Therefore, this review discusses grasshopper‐based foods in terms of their nutritional value, environmental benefits, and conformance with requirements for being halal. This includes exploring Islamic perspectives, Quranic references, and Hadiths, while highlighting the need for deeper evaluation and guidance from Islamic scholars regarding the permissibility of various grasshopper species. This also involves reviewing challenges and opportunities regarding halal certification, consumer acceptance, and market access, especially within predominantly Muslim countries. Grasshoppers align with sustainability goals due to their high feed conversion efficiency, low greenhouse gas emissions, and minimal land and water usage. Additionally, their acceptance in halal markets provides a culturally appropriate alternative protein source. With value addition in food processing and effective consumer education, grasshopper‐based products could become mainstream staple foods both in the halal markets and globally for a sustainable and culturally appropriate solution to future food security challenges.

## 1 Introduction

With the world's population growing rapidly and increasing the demand for sustainable and healthy food, edible insects, particularly grasshoppers, have become increasingly used as a viable alternative source of protein. Grasshoppers are one of the important sources of protein in various parts of the world. Because of their ecological benefits, high nutritional value, and potential contribution to improving food access, their usage has increased. Although entomophagy, or insect consumption, is a common practice in many regions of Africa, Asia, and Latin America, it is now also being considered in Western countries. This is due not just to the nutritional value of insects themselves, but also to the growing need for alternative modes of food production as the livestock sector becomes increasingly unsustainable (Dossey 2013; Suresh et al. 2023).

Among insects, grasshoppers have a high protein content, a low environmental impact, and a higher feed conversion ratio (FCR) than classic livestock (Das and Mandal 2013). Considering the rising global interest in sustainable food systems, grasshoppers have emerged as one of the most promising alternative sources of animal protein when compared to traditional sources such as beef, poultry, and fish. The world's food system accounts for a large percentage of greenhouse gas emissions, while the livestock sector is responsible for deforestation, and greater water use and methane production (Sabri et al. 2023). Grasshoppers, however, being ectothermic animals, require much smaller portions of feed and water and generate much lower amounts of greenhouse gas emissions measured per kilogram of protein produced making them an environmentally friendly protein option (Abdul‐Talib and Abd‐Razak 2013). In addition, they can be reared on organic by‐products, which diminishes the ecological impact of food production (Suresh et al. 2023).

Notwithstanding the benefits, insect consumption, more so in the form of an insect protein‐containing product, particularly burgers, meets the cultural and religious needs of Islamic consumers. While insect protein burgers provide a recognizable format for introducing grasshopper‐based foods, their application need not be limited to this form. Grasshopper protein can be incorporated into a variety of food products, including protein bars, pasta, and soups, broadening its appeal and market penetration. Ensuring these products meet halal certification standards is essential for their adoption in Muslim‐majority markets, where dietary laws significantly influence consumer choices. Furthermore, the inclusion of insect protein in diverse food products beyond burgers better aligns with Islamic dietary diversity and allows for greater accessibility within halal food markets.

The notion of halal, or what is permissible under Islamic law, deeply affects food choices for a significant percentage of people around the globe. In Islam, food habits are determined by the principles of halal, or food that is permissible, and haram, or food that is forbidden, according to both the Quran and the Hadiths. Halal refers to what is permissible under Islamic law, while haram denotes forbidden items.

In this context, it is important to clarify the distinction between grasshoppers and locusts within Islamic dietary law. Locusts are a specific type of grasshopper belonging to the Acrididae family that can enter a swarming (gregarious) phase under certain environmental conditions, such as crowding or food scarcity. Locusts are explicitly mentioned in Hadith as halal and were consumed by the Prophet Muhammad (peace be upon him) and his companions. While locusts and grasshoppers share many biological characteristics, not all grasshoppers are locusts, and therefore the halal status of grasshoppers in general is subject to scholarly interpretation. Some scholars extend the permissibility of locusts to grasshoppers due to their close biological relationship, while others advise caution. In this review, the term *locust* is used when referring to insects explicitly considered halal in Islamic texts, and *grasshopper* is used in ecological, nutritional, and sustainability contexts.

The growing demand for more creative and permissible foods, due to an estimated global Muslim population of more than 1.8 billion in 2024, drives significant growth in the halal food sector. Products incorporating grasshoppers would provide a valid solution for such markets, while also contributing to solving some problems related to global food sustainability (Boukid et al. 2023).

Moreover, nutritional properties of grasshoppers make them a potential option for health‐conscious individuals. Grasshoppers are rich in quality protein, essential amino acids, vitamins, and minerals and, therefore, can be a nutritious alternative to traditional meat products (Blásquez et al. 2012).

Grasshoppers have up to 68% protein by dry weight, as well as large amounts of fiber, fats, and micronutrients such as iron, zinc, and magnesium (Ademolu et al. 2010). The grasshopper contains a protein level equivalent to that of lean meats and contains favorable polyunsaturated fatty acids (PUFA) (Priyatnasari et al. 2024).

These nutritional benefits, plus their ecological sustainability, make the grasshopper (locust) a promising candidate for the halal food industry (Agbidye et al. 2009).

New food products developed featuring grasshopper protein have entered the market. Companies and researchers are testing new approaches to marketing insect‐based foods to mainstream audiences. Examples include the grasshopper hamburger, in which grasshopper protein is combined with other plant‐based ingredients into a nutritious, flavorful, and environmentally sustainable alternative to a beef hamburger. Nevertheless, the entry of these products into the halal food market requires a cautious assessment of religious provisions, consumers' acceptance, and procedures for halal certification (Cerritos 2009; Suresh et al. 2023).

The entire procedure of achieving halal certification for insect‐based food products requires verification from sourcing to processing to ensure the production chain aligns entirely with Islamic principles. This includes verifying how grasshoppers are grown, collected, and processed to ensure no possibility of cross‐contamination with haram substances. Inspections and transparency of the entire process will be required for certification (Akhtar and Isman 2017). Additionally, transparency and consumer education and awareness campaigns will be useful in overcoming initial cultural hesitancy to consume insects, especially in Muslim‐majority regions that may not be familiar with entomophagy (Wu et al. 2014).

This paper discusses the halal status of food products made from grasshoppers (locusts) in the light of their nutritional values, ecological benefits, and religious issues related to their consumption. By examining the relationship between sustainable food and Islamic prescriptions for diet, this article attempts to present a more thorough review of products containing grasshopper (locust) or locust‐derived ingredients’ potential to become a halal, healthy, and ecologically sustainable source of protein for the world's growing population.

## 2 Global Halal Market Trends

The global halal market has experienced strong growth in recent years, due to an increasing Muslim population and greater awareness of halal principles worldwide. The market's expansion is not limited to food; it includes pharmaceuticals, cosmetics, and fashion aligning with Islamic guidelines. With over 1.8 billion Muslims globally, halal products serve a large consumer base that prioritizes religious adherence alongside quality and safety. In regions such as the Middle East, Southeast Asia, and South Asia, halal certification is not just a preference but a necessity for market success (Akbar et al. 2023).

This growth is also supported by non‐Muslim consumers who perceive halal products as more ethical, cleaner, and more sustainable. Halal certification ensures that food and beverages meet stringent quality standards and are free from contaminants and harmful additives. Non‐Muslim‐majority countries, including the United States, Canada, and parts of Europe, are seeing increased demand for halal‐certified goods due to these perceived benefits, creating a successful global market valued at over $2 trillion (Izberk‐Bilgin and Nakata 2016).

Governments and businesses are increasingly investing in halal infrastructure, certification bodies, and specialized export hubs. The halal market is also leveraging digital platforms, with e‐commerce channels and apps dedicated to halal products seeing great usage. These platforms help bridge the gap between producers and consumers, making halal goods more accessible. Additionally, halal tourism has emerged as a significant trend, with destinations offering Muslim‐friendly services, from prayer facilities to halal‐certified dining options (Sani and Dahlan 2015).

### 2.1 The Halal Meat Market

The halal meat market, the most important part of the global halal economy, is seeing robust growth driven by increasing demand from both Muslim and non‐Muslim consumers. Valued at over $1 trillion, this market segment is bolstered by the expanding Muslim population and greater awareness of halal dietary practices. In recent years, climate change and sustainability have become important factors for many consumers, including those in Muslim‐majority countries (Ali, 2015). Among Arab Muslim consumers, awareness of environmental sustainability is gradually increasing, particularly among youth and urban populations, although halal compliance remains the primary determinant of food choice (Abbasi 2024; Hassan et al. 2022). Countries like Saudi Arabia, Indonesia, and Malaysia lead global consumption, while regions such as North America and Europe are emerging as significant markets not only due to growing Muslim populations but also due to some increased interest from non‐Muslim consumers. However, evidence suggests that while some non‐Muslim consumers associate halal with cleanliness and humane treatment, their actual understanding of halal principles remains limited (Ayyub 2015; Lever and Miele 2012). In a UK study, over 15% of non‐Muslim respondents reported occasionally purchasing halal meat, yet only a minority demonstrated accurate knowledge of its religious and ethical foundations (Lipka 2017). These concerns may contribute to increased interest in environmentally friendly foods, including insects, but only when aligned with religious, cultural, and sensory expectations. Key influencing factors in Muslim dietary decisions include halal certification, hygiene, affordability, animal welfare, and increasingly, environmental ethics tied to Islamic principles.

Halal meat production emphasizes humane animal treatment, cleanliness, and compliance with Islamic law, which requires animals to be healthy, slaughtered in the name of Allah, and drained of blood. These principles align with contemporary consumer trends emphasizing food safety, ethical sourcing, and environmental sustainability. As a result, halal meat is becoming increasingly appealing to consumers beyond religious boundaries, particularly those seeking clean products (Riyaz 2023).

Major exporters of halal meat, such as Brazil, Australia, and New Zealand, have developed stringent halal certification systems to meet the needs of Muslim‐majority markets. Meanwhile, technology is having a transformative role, with blockchain being used to ensure traceability and transparency in the halal meat supply chain (Orsi et al. 2019). This increases consumer trust and strengthens market potential. Additionally, the rise of halal‐certified processed meat products, such as sausages, burgers, and ready‐to‐eat meals, is diversifying the market and attracting younger demographics (Azam and Abdullah 2020).

## 3 Grasshopper‐Protein Market Trends and Sustainability

Insect‐based proteins are becoming a viable solution to address global food security challenges while meeting the growing demand for environmentally sustainable dietary options. Grasshoppers (locusts), which are permissible under Islamic dietary laws, are becoming important contenders in the halal protein market.

Grasshoppers and other insects offer significant ecological benefits compared to traditional livestock farming. They require less feed, water, and land, and emit fewer greenhouse gases, making them a more environmentally friendly option. However, it is important to note that sustainability comparisons between different protein sources should still consider factors like feed type, FCRs, and overall environmental impact. For example, crickets only need 1.7 kg of feed to produce 1 kg of body mass, while cattle require 8 kg for the same yield. Fish, with a nearly 1:1 FCR, also have advantages, although their farming relies on fishmeal and fish oil, which can contribute to overfishing (Alexander et al. 2017; Van and Oonincx 2017). Furthermore, cattle can convert fibrous, non‐human‐edible biomass such as grass into protein, which complicates direct feed conversion comparisons. These factors emphasize the need for a comprehensive approach when evaluating the sustainability of different protein sources, ensuring that all aspects of production and environmental impact are considered.

While insects, such as black soldier fly larvae, can be reared on organic by‐products such as those from the food industry, it is important to consider the halal implications of using certain types of feed. Specifically, food waste that may include non‐halal ingredients or contaminants could raise concerns about impurity (najis) under Islamic dietary laws (Riyaz 2023; Van and Oonincx 2017). Therefore, it is important to ensure that any food by‐products used in insect farming are carefully selected to comply with halal standards, ensuring that the feed sources do not have any issues for halal certification. For grasshoppers, the use of organic by‐products as feed has not been widely explored or documented. However, should this method be considered for grasshopper farming, the halal status of the feed would need to be carefully evaluated to ensure compliance with dietary requirements (Abdul‐Talib and Abd‐Razak 2013).

The growing interest in insect‐based protein is partly driven by the global population's projected increase to 9.7 billion by 2050 (Nirmal et al. 2024). Meeting the nutritional requirements of the growing global population requires innovative protein sources, as traditional livestock farming cannot sustainably meet the increasing demand due to its resource‐intensive nature. Alternative proteins, such as insects, can effectively address this demand, providing a diverse, sustainable, and scalable solution. Insects, with their short reproductive cycles, high feed conversion efficiency, and minimal space requirements, offer significant advantages. They can be farmed in various settings, from large‐scale industrial facilities to small urban spaces, making them accessible in both developed and developing regions (Akhtar and Isman 2017).

Muslim‐majority countries such as Malaysia and Indonesia are at the forefront of integrating insect protein into halal‐certified food products. These nations are investing in research and development to create innovative food items such as burgers, protein bars, and snacks that comply with halal standards. The aim is to mainstream insect protein in Muslim markets while also appealing to environmentally conscious non‐Muslim consumers. However, research indicates that halal awareness remains low among non‐Muslim consumers: 47.5% of a surveyed sample reported having no knowledge about halal, and 44.2% said they had only some knowledge (Wilkins et al. 2019). This limited awareness underscores the need for targeted consumer education to support market expansion into non‐Muslim segments. Transparency in sourcing, processing, and halal certification is important to building trust and ensuring compliance with Islamic dietary laws.

Globally, entomophagy, the practice of consuming insects, is already a dietary norm for over 2 billion people across 130 countries (Raheem et al. 2019). Regions with strong entomophagy cultures, such as Thailand, Mexico, and the Democratic Republic of Congo, regularly feature insects like crickets, grasshoppers, locusts, mealworms, and caterpillars in their diets (Boukid et al. 2023; Cerritos 2009; Khan 2018). These insects are consumed in various forms, including raw, roasted, and fried. Besides the previously mentioned benefits, such as being rich in essential amino acids and vitamins, they, in addition, have chitin in their exoskeleton that provides dietary fiber that supports gut health and digestion (Govorushko 2019).

Despite these benefits, cultural resistance remains a significant barrier, particularly in Western countries where the idea of consuming insects has traditionally been met with skepticism. However, this perception is changing due to increased awareness of the environmental and nutritional advantages of edible insects. The demand for sustainable protein sources has led to the development of insect‐based food products such as protein bars, insect flour, and burgers. Companies such as Entomophagy Farms and Aspire Food Group are leading the commercialization of edible insects, making them more appealing and accessible to Western consumers (Akhtar and Isman 2017).

One of the important drivers for the growth of the insect‐based food industry is its potential to mitigate the environmental impact of conventional livestock farming. The livestock industry is a major contributor to greenhouse gas emissions, deforestation, and water scarcity. On the other hand, insects have a far smaller ecological footprint. Their good FCRs and ability to thrive on organic by‐products make them an environmentally friendly alternative. For example, when compared on an equal weight basis, rearing insects requires significantly less water and land than cattle and poultry, making them a more sustainable protein source (Van and Oonincx 2017). The halal‐certified insect‐protein market represents a special intersection of sustainability and religious adherence. Muslim‐majority countries are not only addressing the nutritional needs of their populations but also contributing to global efforts to reduce the environmental impact of food production. By ensuring halal compliance and promoting the ecological benefits of insect protein, this market has the potential to bridge cultural and religious divides, reaching a broad audience that includes environmentally conscious individuals and those seeking ethical food choices.

Reports indicate that the global edible insect market is reaching the stage where it is ready for significant growth, with projections exceeding $8 billion by 2030. This growth is due to technological advancements in food processing, which enhance the palatability and appeal of insect‐based foods (Jameel 2023). Awareness campaigns highlighting the ecological and nutritional benefits of insects are also breaking down cultural barriers. As a result, insect‐based products are becoming increasingly accepted in both Muslim and non‐Muslim markets (Khan 2018).

Insect farming is not only a sustainable solution but also a versatile one, with applications extending beyond human consumption. Insects can be used as animal feed, contributing to a more efficient and sustainable livestock industry. The potential for insects to convert organic by‐products into nutritious food aligns with global efforts to adopt circular economic principles, further emphasizing their role in sustainable food systems (Van Broekhoven et al. 2015).

As demand for ethical and sustainable protein sources grows, the insect‐based halal market has the potential to have a significant role in the global food industry. By overcoming cultural resistance, ensuring halal certification, and highlighting both ecological and nutritional benefits, this sector can meet the needs of an expanding and diverse population. Insects offer a unique combination of sustainability and cultural alignment, positioning them as a forward‐thinking solution for the future of food.

## 4 Nutritional Value of Grasshoppers

Grasshoppers, like many edible insects, provide a good source of nutrients, making them an important alternative source of protein for human consumption. Their high protein content along with essential fats, fibers, vitamins, and minerals is why they are highly recommended as nutritious food sources that can replace traditional animal proteins such as beef, chicken, and fish (Das and Mandal 2013). This section aims to provide detailed nutritional information about their potential contributions to human health (Dossey et al. 2016).

### 4.1 Protein Content

Grasshoppers are a rich source of protein, with their protein content ranging between 43% and 77% on a dry‐weight basis, depending on the species and environmental factors (e.g., diet and harvesting methods). Some studies have found that grasshoppers can provide up to 68% protein by dry weight, making them a highly protein‐dense food source (Patel 2019). In comparison to traditional animal sources like beef and chicken, grasshoppers offer a similar or even higher protein content. For example, *Locusta migratoria* (migratory locust) contains approximately 50%–60% protein when dried. This makes grasshoppers useful, especially in regions where animal protein is scarce or expensive (Ademolu et al. 2010).

### 4.2 Essential Amino Acids

Grasshoppers are quite high in branched‐chain amino acids (BCAA): leucine, isoleucine, and valine, which have physiological implications in muscle protein synthesis and repair. Lysine is an essential amino acid that aids in calcium absorption, immune function, and collagen production (Bukkens 1997). They also contain substantial amounts of methionine and cysteine, sulfur‐containing amino acids that are necessary for detoxification and maintenance of healthy skin, hair, and nails (Agbidye et al. 2009).

In addition to their complete amino acid profile, grasshopper proteins have demonstrated high digestibility, ranging from 77% to 98% depending on species and processing methods (Yi et al. 2013). This is comparable to or even higher than the digestibility of many plant proteins such as soy (78%–85%) and approaches that of animal proteins like beef (94%) and eggs (97%) (WHO 2007). Grasshoppers also lack antinutritional factors such as phytates, allowing for improved amino acid absorption and making them a highly bioavailable protein source.

### 4.3 Fat Content

Grasshoppers contain moderate fat content, often between 4% and 20% depending on the species. Their fat content is generally less than that of normal meats, but grasshoppers contain relatively high PUFA. On average, grasshoppers provide 1.2–2.3 g of PUFA per 100 g of dry weight, comprising approximately 35%–50% of their total fat content (Rumpold and Schlüter 2013). These include essential fatty acids such as those that are omega‐3 and omega‐6 (Das and Mandal 2013). These are known to benefit cardiovascular health and aid in the reduction of inflammation and brain functions (Siddiqui et al. 2023). In comparison, beef provides about 0.1–0.2 g of PUFA per 100 g, chicken about 1.2 g/100 g, and oily fish such as salmon offer around 2.5–3.5 g/100 g (Drewnowski et al. 2024). With them being low in cholesterol, grasshoppers are a better fat source than red meats such as beef and pork that are high in saturated fats and cholesterol (Agbidye et al. 2009).

### 4.4 Dietary Fiber Content

Unlike conventional animal proteins, grasshoppers are a source of dietary fiber with their chitin content, which is one of the components of their exoskeleton. The digestion of chitin by humans is still under investigation, but it is generally credited with benefits for gut health and digestion. Fiber in grasshoppers ranges from 3% to 12% and is considered a prebiotic that promotes gut biodiversity (Bukkens 1997). This sets insects apart from other protein sources that do not contain dietary fiber (Agbidye et al. 2009). While chitin contributes to dietary fiber and has functional food properties, it may reduce protein digestibility due to its rigid structure. Processing methods such as de‐chitinization or fine grinding can improve digestibility and nutritional value. Balancing chitin's benefits with its potential drawbacks is essential for optimizing the formulation of insect‐based foods (Bonomini et al. 2024).

Although the rate of protein degradation in the small intestine can be metabolically adjustable, the need for a supply of amino acids is important to support post‐absorptive nitrogen transport. This is particularly true since many of the essential and BCAA have been shown to stimulate muscle protein synthesis (Ademolu et al. 2010). Because of the amino acid composition of grasshoppers, they qualify as a source of complete protein as they contain all nine essential amino acids that are indispensable for human health (Bukkens 1997). They have an amino acid profile similar to that of beef, chicken, or fish (Banjo et al. 2006).

### 4.5 Grasshoppers as Animal Feed

In addition to their use in human food, grasshoppers are increasingly explored as an ingredient in animal feed, particularly for poultry and aquaculture. Their high protein content and amino acid profile make them a viable alternative to fishmeal and soybean meal. In Pakistan, initiatives have turned locusts into poultry feed to manage swarms and improve feed availability (Arshad et al. 2022). From an Islamic perspective, feeding animals with insects like grasshoppers is generally permissible, provided the feed does not contain haram substances or cause harm to the animals or humans who consume them (Egonyu et al. 2021; Khan 2018). However, there is ongoing scholarly debate, and fatwas regarding animal feed ingredients vary. As the industry expands, formal halal certification protocols for insect‐based feed may need to be standardized to guide producers.

## 5 Comparison of Conventional Protein Sources

When compared to traditional protein sources like beef and chicken, grasshoppers offer a competitive amino acid profile. For example, studies have shown that grasshoppers contain more tryptophan (important for serotonin production) and leucine (essential for muscle repair) than many plant‐based protein sources, such as soybeans (Das and Mandal 2013). Grasshoppers also provide higher protein digestibility (ranging from 77% to 98%) than certain plant proteins, making them a better option for meeting protein requirements, particularly in protein‐deficient populations (Agbidye et al. 2009; Banjo et al. 2006; DeFoliart 1991).

Compared to plant‐based protein sources such as soybeans, lentils, and chickpeas, grasshoppers provide a superior protein concentration and a more complete amino acid profile. For instance, grasshoppers contain approximately 60%–77% protein on a dry weight basis, while soybeans typically contain around 36%–40% (Van Huis 2013). Grasshopper protein is also richer in essential amino acids like leucine, lysine, and valine, which are sometimes limiting in plant proteins. Furthermore, grasshoppers offer higher bioavailability and digestibility, with digestibility values reported between 77% and 98%, compared to 65%–90% for most legumes. In addition to protein, grasshoppers provide key micronutrients like iron, zinc, and vitamin B12, which are either absent or poorly absorbed from plant‐based sources (Rumpold and Schlüter 2013).

### 5.1 Feed Conversion Ratio

Grasshoppers are among the most efficient eco‐eatables when considering feed conversion. The ratio represents conventionally raised livestock with respect to the amount of feed it takes to produce a fixed amount of body mass. Many studies have found that edible insects require markedly less feed than conventionally raised livestock to produce comparable amounts of protein (Cerritos 2009). This efficiency means that grasshoppers require fewer agricultural inputs—such as water and land—which ensures their farming is less harmful to the environment. For example, crickets require only 1.7 kg to produce 1 kg of body weight, while cattle need up to 8 kg of feed for the same amount of meat (Alzeer et al. 2018).

Grasshoppers are among the most efficient edible insects in terms of feed conversion, requiring significantly less feed compared to conventionally raised livestock. While broilers (chickens) and fish have efficient FCRs, with broilers needing around 1.7 kg of feed to produce 1 kg of body mass, insects like grasshoppers still offer an environmentally friendly alternative due to their ability to thrive on fewer agricultural inputs, such as water and land (Alzeer et al. 2018). Grasshoppers also benefit from shorter reproductive cycles and the ability to be farmed in stacked, modular systems such as vertical insect farms in diverse environments—allowing for production in urban or space‐limited areas. This efficiency not only reduces ecological impact but also opens opportunities for scalable farming in resource‐constrained settings. However, actual land use and sustainability outcomes may vary based on farming design and scale. (Bao et al. 2024).

### 5.2 Water use

Grasshopper farming has a lower water footprint than livestock farming. Water usage is especially of concern in food production worldwide, particularly in the arid and semi‐arid regions where water scarcity is a growing problem (Gahukar 2016). Grasshoppers require far less water for rearing than cattle and poultry. For instance, beef is often of concern in water usage comparisons because it is one of the most water‐intensive proteins to produce, and it takes about 15,000 L of water to produce 1 kg of beef (Bhagat et al. 2020). Poultry also require considerable amounts of water compared to the lesser (but not well documented) quantities for 1 kg of insect, including grasshoppers; therefore, grasshoppers could be an adequate source of protein in regions that are short of water (Bukkens 1997).

### 5.3 Use of Land

Grasshopper farming requires much less land than livestock farming. Many negative consequences, including deforestation, habitat degradation, and biodiversity loss, are caused by livestock agriculture due to a requirement for substantial land to graze animals and plant feed crops. While chickens also benefit from more efficient land use, grasshoppers can be farmed in much smaller spaces and even vertically, maximizing land efficiency (Das and Mandal 2013). The reduction in land requirements again helps ameliorate various environmental issues like soil erosion and land degradation (Banjo et al. 2006; Gahukar 2016).

### 5.4 Low Greenhouse Gas Emissions

Perhaps the most favorable environmental benefit of grasshopper farming is its low contribution to greenhouse gas emissions. The livestock sector is a major source of methane, a potent greenhouse gas, as well as carbon dioxide and nitrous oxide emissions. According to the FAO, livestock production is responsible for 14.5% of all human‐induced greenhouse gas emissions, although this figure may vary regionally, and substantial progress has been made in methane reduction efforts with ruminant farming. (Cerritos 2009). On the other hand, grasshoppers and other insects produce almost no greenhouse gases (Gahukar 2016). For example, producing 1 kg of beef protein emits about 60 kg CO_2_‐equivalent, pork emits around 7 kg CO_2_‐equivalent, and chicken about 6 kg CO_2_‐equivalent. In contrast, grasshoppers emit as little as 1–2 kg CO_2_‐equivalent per kg of protein, making them significantly more climate‐friendly (Van Huis and Oonincx 2017; Gahukar 2016). Thus, grasshoppers would be a climate‐friendly option for proteins that can help combat global warming (Akhtar and Isman 2017).

### 5.5 Cultivation and Environmental Considerations

While grasshoppers represent a nutritionally valuable protein source, large‐scale cultivation—particularly of locust species’ raises environmental concerns due to their potential to cause crop destruction. However, recent studies highlight that non‐swarming grasshopper species may be more suitable for controlled farming. Sustainable rearing systems such as vertical farming, climate‐controlled chambers, and netted indoor facilities are being explored as alternatives that minimize ecological risk while supporting consistent production (Gotham and Song 2013). These methods also align with halal processing requirements and traceability in the food supply chain.

## 6 Potential Health Benefits of Grasshoppers Consumption

The high nutritional value of grasshoppers translates into numerous health benefits, especially when part of a well‐balanced diet. Here are a few selected health benefits:

- *Muscle growth and repair*: Grasshoppers are a good protein source for enhancing muscle growth and repair, as they provide all the essential amino acids. However, like other protein sources, they may have a rate‐limiting essential amino acid, which can vary depending on the species and diet of the insect. Studies suggest that methionine and lysine are often the limiting amino acids in many edible insects, including grasshoppers (Bukkens 1997). Despite this, grasshoppers are rich in BCAA, particularly leucine, which plays a crucial role in triggering muscle protein synthesis. This high leucine content makes them beneficial for maintaining lean body mass and facilitating recovery after exercise or injury (Orsi et al. 2019). Ensuring a well‐balanced diet or complementing insect protein with other protein sources rich in the limiting amino acids can optimize their nutritional benefits.
- *Cardiovascular health*: Rich in unsaturated fats of both the omega‐3 and omega‐6 fatty acid families, these are undoubtedly useful for the heart. Pancreatic benefit is seen through inhibited release of noxious inflammatory substances. These beneficial fats have been found to lower blood pressure, reduce inflammation, and improve the lipid profile, thus minimizing the risks for heart diseases. Due to their low cholesterol contents, grasshoppers help in promoting heart health and provide a healthier alternative to red meat with their better fatty acid composition (Gahukar 2016; Agbidye et al. 2009).
- *Weight control*: Grasshoppers can aid in weight management due to their high protein content and moderate fat content. Protein contributes to a feeling of fullness, which can help reduce overall calorie intake and promote healthier eating habits. Additionally, the fiber content in grasshoppers may support digestion and help regulate blood sugar levels, further supporting weight maintenance and metabolic health (Bukkens 1997).
- *Gut health*: Chitin works as a prebiotic. This process can help good bacteria thrive in the gut. With a healthy gut microbiome, optimal digestion, immune system function, and even mental health benefit. Preliminary results support chitin possibly being important for gut health and nutrition absorption improvements, but more studies are needed to clarify its benefits (Gahukar 2016).
- *Bone and immune health*: The presence of micronutrients in grasshoppers includes iron, zinc, and magnesium, all of which are required for various functions in the body. Iron is needed for hemoglobin and myoglobin production and the prevention of anemia, zinc is needed for immune support and wound healing, and magnesium is needed for bone health, muscle function, and nerve signaling (Akhtar and Isman 2017).

## 7 Halal Dietary Laws for Insect Consumption

The concept of halal (permissible) in Islam governs not only what food Muslims are allowed to consume but also how it is prepared and sourced. These dietary guidelines, rooted in the Quran and Hadith (the sayings and practices of the Prophet Muhammad [peace be upon him]), provide clear instructions regarding permissible and forbidden foods. Insects, as a category, are generally considered non‐halal, with some exceptions. Locusts are seen as permissible (halal) in Islamic dietary law, based on specific Hadith. Locusts share this status, being explicitly mentioned as halal in various Islamic texts, which sets them apart from many other insect species that are typically avoided (Abdul‐Talib and Abd‐Razak 2013; Regenstein et al. 2003). While locusts are considered permissible in Islam, other insects may not be, and opinions on their permissibility can vary. It is important to consult with halal certification bodies to clarify the acceptability of specific insect species.

### 7.1 Understanding Halal Food Requirements in Islam

Halal applies to the description of foods that are allowed according to Islamic law, as well as how it is prepared. Haram refers to what has been strictly forbidden. Principal among the sources of halal dietary laws is the Quran, enhanced by Hadith, plus the input from Islamic scholars pertaining to the topic.

Halal food is based on the following principles:

- *Slaughter of animals*: The slaughter of living animals falls under halal by the law of prescription in Islam. Animals must be slaughtered in the name of Allah by a Muslim. Religious slaughter must be done based on the principle of causing minimal suffering of the animal (Alzeer et al. 2018).
- *Forbidden food*: Certain animals are already forbidden (haram) under Islamic dietary laws. Prominent examples include carnivorous animals and animals that are slaughtered without adherence to the prescribed method. Blood, intoxicants, and the sacrifices made to other deities are also prohibited (Riyaz 2023).
- *Purity and cleanliness*: Halal food must be pure, clean, and devoid of harmful materials. Any cross‐contamination or mingling with haram materials makes the food non‐halal.

In many cultures and religions, insects are generally classified as impure or unsuitable for consumption, often due to concerns about hygiene, religious beliefs, or the perception of insects as pests. These views are particularly common in regions where insects are not traditionally part of the diet, but Islamic law makes a clear exception regarding certain insects, such as locusts. This distinction has been backed by Islamic scholars based on the interpretation of religious texts with specific rulings about insects such as locust (Dossey 2013; Lever and Miele 2012).

### 7.2 Islamic Religious Texts on the Permissibility of Grasshoppers/Locusts

In Islamic dietary law, the permissibility of locust can be traced through both the Quran as well as the Hadiths. Unlike insects in general, which are regarded as impure and haram, locusts have been specifically deemed halal (El‐Mallakh & El Mallakh, 1994).

It is narrated in a Hadith that:

*“Two kinds of dead animals and two kinds of blood are made lawful for us: fish and locusts, and liver and spleen.”*

—Sunan Ibn Majah 3314

This Hadith is noteworthy in that it explains that, while dead animals are ordinarily forbidden from consumption because they were not slaughtered in accordance with Islamic law, exceptions are made in the case of locusts and fish. Locusts are explicitly mentioned as permissible in Islamic texts. Although locusts and grasshoppers both belong to the family *Acrididae*, not all grasshoppers are locusts. Locusts represent a specific phase of certain grasshopper species that exhibit swarming behavior under particular environmental conditions. The permissibility of grasshoppers in general is not explicitly stated in Islamic texts and remains subject to scholarly interpretation, often based on their biological similarity to locusts.

Another Hadith narrated by Abdullah ibn Umar acknowledges that the Prophet himself used locusts for his dietary needs:

*“Locusts were eaten by us during the lifetime of the Messenger of Allah (peace be upon him).”*

—Sahih Muslim 1952 (Siddiqui, 1976).

This Hadith further establishes that locusts were eaten during the lifetime of the Prophet (PBUH) and were regarded as neither impure nor forbidden.

In addition to these Hadiths, the scholars refer to Surah Al‐A'raf (7:157), which explains that Muslims should eat food that is lawful and wholesome (tayyib). With locust being mentioned in the Hadiths, they fall within the category of permissible and wholesome food.

### 7.3 Scholarly Opinions and Fatwas on Eating Insects

While the Quran and the Hadiths provide general guidance, the interpretation of halal dietary laws in specific cases, such as the consumption of insects, often depends on the rulings of Islamic scholars and fatwas (legal opinions). When it comes to insects, scholars have examined religious texts and have generally agreed on the permissibility of consuming locusts, and the permissibility of other grasshopper species remains subject to scholarly interpretation (El‐Mallakh & El‐Mallakh, 1994; Shrivastava et al. 2019).

#### 7.3.1 Scholarly Consensus

A majority of scholars across the various schools of Islamic jurisprudence, both Sunni and Shia, agree that locust is halal based on the Hadiths mentioned above, which explicitly states that locust may be eaten, dead or alive, with no mandated procedure for slaughter. Other insects are generally considered haram.

For example, in the Hanafi school of thought, which is one of the major Sunni Islamic legal schools, locusts are considered halal because the Prophet Muhammad (peace be upon him) and his companions consumed them (El‐Mallakh & El Mallakh, 1994). Unlike other schools that permit a broader range of seafood, Hanafi jurisprudence typically permits only fish, excluding sea insects like shrimp and crabs. This conservative interpretation also extends to other non‐fish sea creatures (Šerifović et al. 2022).

The Maliki and Shafi'i Sunni schools of thought also permit the consumption of locust based on similar reasoning. They hold that since locust is mentioned as an exception to the general prohibition against consuming dead animals, they are lawful to eat without the need for ritual slaughter (zabiha) (Dossey et al. 2016).

In Shi'ism, there is a clear rejection of locusts as halal in most cases. Shi'a scholars, particularly the Jafari school, do not consider locusts as permissible. The reasoning behind this is rooted in the Shi'a interpretation of Islamic jurisprudence, which does not recognize locusts as a lawful food. In Shi'a law, for an animal to be halal, it must meet specific criteria, such as being a land animal that chews the cud and has split hooves, a sea animal explicitly allowed (like fish with scales), or a creature clearly permitted in authentic Shi'a Hadith. Locusts do not meet these conditions and are not included in the list of explicitly lawful animals in Ja'fari texts; thus, they do not fulfill these requirements (Tajudeen 2020).

#### 7.3.2 Fatwas Regarding Insect Consumption

Numerous fatwas have been issued over the years confirming the permissibility of eating locust. One example is a fatwa issued by the Islamic Fiqh Council, a body of scholars under the Organization of Islamic Cooperation (OIC), which reviews contemporary issues in Islamic jurisprudence. The Council reviewed the Hadiths and scholarly interpretations, concluding that locusts are halal for Muslims to consume. This conclusion stems from the references to locusts in the Hadiths of Prophet Muhammad (peace be upon him), where he and his companions are said to have consumed locusts during their travels (Palupi et al. 2024). However, it is important to note that while the Hadiths predominantly use the term “locust” (Arabic: جراد, “jarad”), rather than “grasshopper,” scholars have extended the permissibility to grasshoppers due to their biological similarity to locusts. Locusts and grasshoppers belong to the same family (Acrididae) and share similar physical characteristics, with the primary difference being that locusts are known for their ability to swarm (Siddiqui et al. 2023). Although the Hadiths focus on locusts, some legal opinions, including interpretations referenced by the Islamic Fiqh Council, suggest that grasshoppers closely related to locusts may also be considered permissible (Shrivastava et al. 2019; El‐Mallakh & El Mallakh, 1994).

Additionally, in regions where locusts are commonly consumed, local scholars have issued fatwas affirming their halal status. In Saudi Arabia, for example, where locust consumption has been a traditional practice, scholars have consistently reinforced the halal status of locust based on the Hadiths that mention them as a permissible food source (Sharawi 2024). The consistent interpretation of these Hadiths, along with the biological similarities between locusts and certain grasshoppers’ species, extends permissibility to those grasshoppers, though this remains subject to interpretation rather than universal consensus. These local fatwas reflect a scholarly view that, in specific contexts, grasshoppers closely related to locusts may also be considered halal due to their comparable characteristics. On the other hand, fatwas concerning other insects often rule them as haram due to their impure nature, lack of clear permissibility in the texts, and cultural associations with filth (Shamsuddin 2024).

However, in exceptional cases of necessity, such as famine or extreme food scarcity, scholars have permitted the consumption of normally forbidden insects as an emergency measure (darurah ضرورة), although locust remains a preferred halal option.

Locusts can be a good source of food for Muslim communities, particularly in regions where they are available abundantly and can be harvested sustainably. With an increasing interest in entomophagy worldwide, the religious acceptance of locusts being halal can thrive in acceptance in halal markets, adding value to sustainable food systems (Lever and Miele 2012; Shrivastava et al. 2019).

#### 7.3.3 Islamic Perspectives on the Halal Status of Grasshoppers/Locusts

In Islamic dietary law, all food is considered permissible (halal) unless explicitly prohibited by the Qur'an or Hadith. Surah Al‐Baqarah (2:173) and Surah Al‐Ma'idah (5:3) outline the key prohibitions, including carrion, blood, pork, and animals not slaughtered in the name of Allah. While locusts are explicitly mentioned as halal in Hadith literature, grasshoppers occupy a more complex and debated space within Islamic jurisprudence. A well‐known Hadith recorded in Sunan Ibn Majah (2007) (Book 27, Hadith 3238) quotes the Prophet Muhammad (peace be upon him) as saying: “*Two dead things are lawful for us: fish and locusts*.” (Majah & Yazid, 2015)

Locusts are also referenced in Surah Al‐A'raf (7:133) among the signs sent to Pharaoh. Taxonomically, both locusts and grasshoppers belong to the Acrididae family; however, not all grasshoppers are locusts, and this distinction has implications for permissibility under Islamic law (Al‐Qaradawi 1999).

Some Islamic scholars support the permissibility of grasshoppers by analogy to locusts, citing their biological similarity and functional interchangeability in certain ecosystems. Others urge caution, noting that early Islamic texts refer specifically to locusts, and extending this ruling to all grasshoppers may require further scholarly consensus. With more than 10,000 grasshopper species identified worldwide, the lack of specificity presents a challenge for consumers and certifying bodies alike.

Moreover, Islamic rulings on food emphasize not only permissibility but also cleanliness, safety, and the avoidance of harm. Therefore, any novel food source, including insects, must be assessed for potential allergenicity, contamination, and ethical sourcing. Consumer acceptance is also shaped by cultural traditions, taste, and familiarity (Song 2010). While locusts show promise as a sustainable and nutritious protein, further religious clarification, ideally through fatwas or position statements from respected Islamic authorities, may be needed to strengthen consumer confidence and support halal market access.

### 7.4 Consumer Acceptance and Cultural Perceptions

The acceptability of locust‐based products in Muslim‐majority regions is influenced by multiple interlinked factors: taste preferences, sensory characteristics, allergenic potential, and most critically, their halal status. Even though some scholarly opinions support permissibility by analogy to locusts, the absence of unanimous consensus across Islamic schools of thought leads to hesitation. Without clear and widely accepted religious rulings, many consumers may remain skeptical about incorporating locusts into their diet, regardless of their nutritional or environmental advantages.

Cultural familiarity plays a significant role in shaping consumer attitudes. In many Muslim‐majority countries, entomophagy—the practice of eating insects—is not a traditional or widespread dietary behavior. This unfamiliarity often leads to discomfort or aversion, particularly when products retain visible insect features. Thus, food product development should prioritize forms that minimize visual cues (e.g., flour or protein powders) and enhance flavor, texture, and aroma using appropriate processing techniques to make the final products more appealing and acceptable (Riyaz 2023).

In addition to cultural barriers, there are also valid health‐related concerns. Locusts, like other insects, may pose allergenic risks, particularly due to potential cross‐reactivity with crustaceans such as shrimp and crab. This underlines the importance of rigorous allergen testing, clear labeling, and consumer education to ensure safe and informed choices (Palupi et al. 2024).

Ultimately, shifting consumer perception will require a multi‐pronged strategy. Religious clarification from trusted scholars, innovative food design, strategic marketing, and education about environmental and health benefits will be essential to making locust‐based products viable in halal markets. When positioned correctly, such products offer a unique opportunity to meet the growing demand for sustainable and culturally appropriate protein sources.

## 8 Potential Contribution to Food Security in Muslim‐Majority Regions

Locusts have the potential to have an important role in improving food security, particularly in Muslim‐majority regions where the demand for halal food is significant. Recent global locust swarms have highlighted both the destructive potential and the untapped utility of locusts. In countries like Pakistan and Saudi Arabia, governments have adopted innovative approaches to manage swarms by converting them into protein sources for both human and animal consumption. While locust swarms threaten food security by damaging crops, they also represent a readily available biomass that, if harvested efficiently and hygienically, can serve as a valuable food resource. Sustainable harvesting must be balanced with environmental and agricultural protection (Sultana et al. 2021).

Several factors make locusts an ideal solution for addressing food security:

- *Meeting protein demand*: Many Muslim‐majority countries, particularly in Africa, the Middle East, and Southeast Asia, are challenged to provide affordable, high‐quality protein sources. Traditional livestock farming in these regions is often constrained by resource limitations, such as water scarcity, poor grazing land, and limited feed availability. Locusts farming offers a viable alternative, providing a high‐protein, nutrient‐dense food source that can be produced with minimal inputs (Ali and Regenstein 2014).
- *Cultural and religious acceptance*: Locusts are considered halal in Islam, making them a permissible and viable protein source for Muslim consumers. Their availability in many regions, combined with religious acceptance, increases their potential adoption in Muslim‐majority countries. Additionally, local locusts farming can reduce reliance on imported meat, mitigating costs and supply chain disruptions (Šerifović et al. 2022).
- *Adaptability to local farming conditions*: Locusts are highly adaptable to arid and semi‐arid regions where traditional livestock farming faces challenges. They require minimal water, with estimates indicating they need far less than conventional livestock like cattle or poultry. Additionally, locusts can be fed local crops or organic by‐products, although care must be taken to ensure feed sources comply with halal standards (Orsi et al. 2019). Their resistance to many livestock diseases further enhances their suitability as a protein source in regions with environmental challenges like drought or water scarcity. Small‐scale locusts farming can be integrated into existing agricultural systems with minimal infrastructure, making it feasible for local farmers. It also offers an opportunity to generate additional income by diversifying agricultural practices (Akhtar and Isman 2017).
- *Economic opportunities*: In addition to addressing food security, locusts farming can provide economic opportunities in Muslim‐majority regions. The production of locusts can be a low‐cost, high‐yield enterprise, particularly for smallholder farmers. By raising locusts, farmers can diversify their income, increase resilience to market fluctuations, and reduce dependence on livestock, which are more resource‐intensive and costly to maintain. Furthermore, the growing demand for alternative proteins, both domestically and internationally, opens export opportunities for locust‐based food products, contributing to local economies (Ali and Regenstein 2014).

## 9 The Halal Certification Process for Insect‐Based Food Products

For locust products to be accepted in Muslim‐majority markets, they must comply with the principles of halal certification (Cerritos and Cano‐Santana 2008; Lever and Miele 2012). The halal certification process for insect‐based foods has some special challenges, including navigating traditional halal guidelines and ensuring transparency throughout the supply chain (Abdul‐Talib and Abd‐Razak 2013).

Halal certification is a process through which food products are certified as permissible for consumption according to Islamic dietary laws. The process typically involves ensuring that the product is free from haram ingredients and that the entire supply chain, from sourcing to processing to consumer sales, adheres to Islamic guidelines (Regenstein et al. 2003).

### 9.1 Key Steps in Halal Certification

- *Ingredient verification*: The first step in halal certification is verifying that all ingredients used in the product are halal. In the case of insect‐based products, it is necessary to show that the insects themselves are permissible. However, other ingredients used in the food product, such as binding agents or flavor additives, must also be evaluated to ensure they meet halal standards (Lever and Miele 2012; Shrivastava et al. 2019).
- *Processing standards*: The halal certification process also examines how the food is processed. This includes ensuring that the equipment used for processing insect‐based foods is not contaminated with haram substances, such as pork or alcohol. Additionally, the storage, packaging, and transportation of the product must comply with halal guidelines, avoiding any cross‐contamination with non‐halal foods (Ali and Regenstein 2014).
- *Inspection and auditing*: Halal certification authorities are responsible for inspecting and auditing facilities where insect‐based food products are produced to ensure compliance with Islamic principles. For locust farming, this includes verifying that the insects are harvested and handled in a way that aligns with halal standards, without engaging in any haram practices (Riyaz 2023).

Regarding humane methods of insect handling, commercial locust farming is still in the process of developing and standardizing ethical and halal‐compliant harvesting methods. Insect slaughter processes need to avoid practices that could be considered inhumane, such as excessive stress or harm during harvesting. Currently, while there are no universally accepted halal slaughter guidelines specifically for insects, emerging practices and research suggest methods such as swift immobilization, chilling, or freezing to reduce movement and metabolic activity prior to killing. These methods aim to minimize potential suffering and ensure hygiene. However, most Islamic jurisprudence has yet to issue specific rulings on insect slaughter, as insects are generally not subject to ritual slaughter requirements in traditional fiqh (Van Huis 2013).

However, much like the debate over the ethical treatment of crustaceans (e.g., lobsters), the question of animal welfare in insects remains less defined due to their biological differences from higher animals (Riyaz 2023). Although insects like locusts are biologically far simpler than mammals, Islamic scholars and halal certifiers may still demand ethical treatment that minimizes harm, as part of upholding the broader principles of mercy and compassion in Islam. As the insect farming industry grows, it is likely that more detailed and formalized standards will emerge to address both halal compliance and humane practices for insect harvesting and processing. While locust farming aligns with halal dietary principles in theory, practical implementation, especially among small‐scale producers, requires structured oversight. Ensuring compliance involves not only using halal‐compliant feed but also maintaining hygienic facilities, avoiding contamination, and receiving appropriate training. Scaling this across decentralized producers would necessitate training modules, inspection systems, and support from halal certifying bodies to maintain standardization (Nizar et al. 2022).

- *Labeling and marketing*: Once certified, the product can carry a halal certification marking, which indicates to Muslim consumers that the food meets the dietary requirements of Islam, and ideally indicates which organization is behind the claim. This certification indication is important for consumer confidence, especially in markets where halal food is highly sought after.
- *Non‐Muslim halal food consumption*: Although halal certification primarily targets Muslim consumers, recent market studies show that non‐Muslim consumers are also purchasing halal products, though motivations vary. In many cases, increased Muslim migration and expanding multicultural urban centers have led mainstream retailers to stock halal items, making them more accessible to all consumers. However, some non‐Muslims actively choose halal foods due to perceptions of cleanliness, animal welfare, and ethical processing standards (Ayyub 2015). For instance, a UK survey found that over 15% of non‐Muslim respondents reported purchasing halal meat occasionally, with health and perceived hygiene cited as key motivators (Lever and Miele 2012). Despite these patterns, confusion about what halal means remains widespread, and more consumer education is needed to support informed choice (Wilkins et al. 2019).

## 10 Challenges and Solutions for Insect‐Based Halal Products

### 10.1 Challenges for Insect‐Based Products

- *Cultural perception of insects*: While locusts are halal, many other insects are considered haram or impure in Islam. Halal certification bodies must ensure that products contain only permissible insects, such as locust, and that no cross‐contamination occurs with non‐halal insects. This is a significant challenge, especially in facilities processing multiple insect types. Currently, the practice of raising multiple insect species in the same facility is not widespread but is an emerging concern as the insect farming industry grows. While it is still more common for farms to focus on single‐species production, such as crickets or mealworms, there is potential for some farms to diversify and farm multiple species simultaneously. In these cases, ensuring that only halal insects, like locusts, are included in products and preventing cross‐contamination with non‐halal species would indeed be a significant challenge. Halal certification bodies would need to strictly monitor these facilities, ensuring that proper segregation measures are in place. These measures could include separate production lines, storage areas, and handling procedures for halal and non‐halal insects (Abbasi et al. 2021). In addition, facilities would need to implement rigorous cleaning and sanitization protocols to avoid contamination. While this issue may not be widespread yet, as the industry evolves, this will become an increasingly important consideration for halal certification bodies to ensure that only permissible insects are used in halal food products. Furthermore, cultural norms in many Muslim‐majority countries heavily favor traditional protein sources such as beef, chicken, and fish, leading to resistance against the idea of consuming insects as part of their diet (Palupi et al. 2024).
- *Regulatory challenges*: Regulatory frameworks for insect‐based food products vary across countries, and in many regions, the guidelines for production, processing, and halal certification are still evolving. While some countries, particularly in the European Union, have established regulations for edible insect production, these guidelines often focus on general food safety rather than specific halal requirements. As a result, halal certification for insect‐based products can be unclear or inconsistent, creating barriers to market entry for producers seeking to meet both halal and food safety standards. An important concern in halal certification is ensuring the cleanliness of the insects, especially regarding the gut contents. Wild‐harvested insects, like locusts, may carry impurities in their digestive systems that could compromise their halal status. With the shift toward farmed insects, this issue could be mitigated by controlling their diet and hygiene, making them more suitable for halal certification (Ali and Regenstein 2014). This transition from wild harvesting to controlled farming practices holds the potential to improve the cleanliness and overall quality of insects for halal markets, helping to address concerns about the impurity of their gut contents.
- *Consumer education and awareness*: Consumer acceptance remains one of the most significant barriers to the adoption of insect‐based halal foods. Psychological aversion, commonly referred to as the “yuck factor,” can deter potential consumers. Even in Muslim‐majority regions where locust has been recognized as halal, the practice of entomophagy is not widespread, and introducing such foods into diets requires careful planning (Kim et al. 2024).
- *Economic viability and market entry*: Producing insect‐based foods at a cost that is competitive with traditional protein sources remains a challenge. Initially, the novelty of these products often results in higher prices because of the research, development, and regulatory processes involved in bringing them to market. Additionally, the lack of established supply chains, specialized farming infrastructure, and processing facilities for large‐scale insect production increases production costs. Moreover, consumer unfamiliarity with insect‐based products and a limited number of suppliers further restricts market access and awareness, which can slow market penetration (Kim et al. 2024). While incremental marketing strategies can help address these barriers by gradually increasing consumer exposure and acceptance, the initial challenges of cost and distribution networks still present significant hurdles in the early stages of market development for insect‐based foods.

### 10.2 Solutions to Address Challenges

Solutions to address challenges are as follows:

- *Emphasizing environmental and health benefits*: Educational campaigns should highlight the environmental sustainability and health benefits of locust‐based foods. Their eco‐friendly and nutritious attributes can appeal to environmentally conscious consumers and younger Muslim audiences who are more open to innovative and sustainable food options.
- *Collaborating with religious scholars and authorities*: Building consumer trust in halal certification necessitates partnerships with religious scholars and authorities. Collaborating with local religious leaders and halal certification bodies can help educate the public on the permissibility and benefits of locust‐based foods. Fatwas and endorsements from respected scholars can lend credibility to insect‐based products, easing consumer concerns about their halal status and encouraging acceptance.
- *Innovative marketing strategies*: Creative marketing strategies can have a significant role in promoting insect‐based foods. Presenting these products in familiar formats, such as burgers, protein bars, and snacks, makes them initially more accessible to hesitant consumers. Organizing free tastings, introducing these products at food festivals, and promoting them through social media campaigns can normalize their consumption. Additionally, aligning marketing messages with Islamic values, such as environmental stewardship (khilafah) and minimizing waste, can further resonate with Muslim consumers.
- *Establishing robust regulatory frameworks*: Developing standardized guidelines for the production, processing, and certification of insect‐based foods is important. Countries such as Malaysia, Indonesia, and Saudi Arabia have begun formulating frameworks for halal certification of insect‐based products. While consistency across halal certification agencies is beneficial, it is also important to acknowledge that halal standards can vary based on religious interpretations. Much like the kosher certification system, which embraces different interpretations of religious law, halal certification can accommodate diverse approaches, as long as they are transparently justified according to Islamic teachings. Expanding these certification initiatives globally, while respecting the diversity of halal practices, will promote consumer confidence and facilitate the integration of grasshopper‐based products into halal markets.
- *Promoting sustainability and innovation*: The environmental benefits of insect farming, such as lower resource requirements and reduced greenhouse gas emissions, should be prominently emphasized. Additionally, promoting the circular economy aspect of insect farming, such as using organic by‐products to rear insects, can enhance their sustainability credentials. Investing in research and development to create cost‐effective production methods and innovative food processing technologies will further make these products more competitive and appealing.
- *Building consumer trust through transparency*: Ensuring transparency in sourcing, processing, and halal certification is necessary for building consumer trust. Halal certification authorities must rigorously enforce compliance with Islamic dietary laws while maintaining high food safety standards. While certifying bodies are not typically responsible for food safety in the traditional sense, they can influence the industry by refusing certification to companies that do not meet the necessary legal standards. Partnerships with trusted religious leaders and endorsements from credible organizations can further enhance consumer confidence. Sharing success stories from regions where insect‐based foods have gained acceptance can also help mitigate skepticism.
- *Economic strategies for market expansion*: To make insect‐based foods more economically viable, governments and private sectors can invest in scaling up production facilities, reducing costs, and improving accessibility. Subsidizing initial production costs and offering incentives to producers can help lower prices, making these foods more competitive with traditional protein sources. Collaboration with established food brands to incorporate insect protein into their offerings can also lead to wider adoption.
- *Consumer education and awareness*: Recent empirical studies confirm that many Muslim consumers remain hesitant to accept insect‐based foods, despite their halal potential. For example, Abbasi (2024) reported that only 18% of surveyed Muslim consumers expressed willingness to try insect‐based food, while 68% were unwilling, citing factors such as unfamiliarity, concerns about religious permissibility, and the visible form of insects in food. However, acceptance increased when products were processed into non‐visible forms like flour or bars and when accompanied by endorsements from halal certifying authorities. Other motivating factors included environmental sustainability, nutritional value, and alignment with Islamic values such as Tayyib (wholesomeness) and stewardship (Khilafah). These findings underline the need for culturally tailored education and transparent religious guidance to support adoption of halal insect‐based foods. Overcoming cultural resistance, regulatory hurdles, and consumer skepticism requires a multifaceted approach encompassing education, collaboration, and innovation.

## 11 Halal Locust Species and Certified Diets

### 11.1 Halal Locust Species

Commonly consumed halal locust species include the following:

- *Locusta migratoria* (migratory locust): Widely consumed globally, this species has a mild, nutty flavor and is often preferred for its versatility in culinary uses (Šerifović et al. 2022).
- *Mecopoda elongata* (brown leaf‐like grasshopper): This grasshopper offers a rich, savory taste, making it ideal for seasoning‐intensive dishes (Sabri et al. 2023).
- *Atractomorpha crenulata* (tobacco grasshopper): Known for its sweet undertones, this species is particularly suited for snacks and sweet‐savory combinations (Sabri et al. 2023).
- *Valanga nigricornis* (Javanese bird grasshopper): A robustly flavored species often used in traditional Southeast Asian cuisines (Aydoğan et al. 2024).

The non‐carnivorous diet of these species ensures their compliance with halal requirements, and their historical and cultural significance enhances consumer acceptance in Muslim‐majority regions.

### 11.2 Rearing Practices for Locust for Halal Compliance

Maintaining the halal status of locust extends beyond their natural characteristics to the conditions of their rearing and processing. Important considerations include the following:

- *Diet*: Locust, like other edible insects, must be fed clean and permissible feeds to ensure their halal status. The feed provided to locusts should consist of organic materials such as grass, leaves, and grains that are free from impurities and haram substances. While locusts can thrive on organic waste, it is essential to avoid any sources that may include non‐halal ingredients such as animal by‐products, alcohol, or other prohibited materials. This is particularly important in farming operations of all sizes, but the risk becomes more pronounced in large‐scale production due to factors such as higher feed volumes, complex supply chains, and shared processing facilities. Large farms often rely on bulk feed procurement, where cross‐contamination with non‐halal ingredients is more likely if not carefully monitored. Additionally, commercial feed suppliers may manufacture multiple types of feed in the same facilities, increasing the chances of unintentional contamination. Automated feeding and bulk storage systems can also mix different feed batches, requiring strict monitoring protocols to maintain halal integrity (Palupi et al. 2024). Regardless of the scale of production, ensuring the use of clean, permissible feeds is essential for maintaining the halal status of the insects, meeting the dietary requirements of Muslim consumers, and upholding the sustainability and safety of the farming process.
- *Controlled rearing environment*: For a facility to meet halal standards, it is necessary to prevent cross‐contamination with haram materials, which includes not only controlling the feed provided to the locust but also ensuring the overall cleanliness of the equipment and environment. Additionally, it may be necessary for farms to implement dedicated halal farming units, where the equipment, storage areas, and processing lines are exclusively used for halal‐certified insect farming to avoid any inadvertent exposure to non‐halal substances (Anjum et al. 2020). These precautions, when properly followed, ensure that the entire production process complies with Islamic dietary laws. Furthermore, inspections and audits by halal certification bodies have an important role in ensuring that these measures are consistently applied.

These practices are not only needed for religious permissibility but also ensure the sustainability and ethical integrity of locusts farming operations (Shamsuddin 2024).

## 12 Sensory Qualities of Grasshoppers

Grasshoppers are nutritionally dense, with attributes that rival or surpass traditional protein sources:

- *Flavor profile*: The natural taste of grasshoppers is often nutty or earthy, with slight variations across species and preparation methods (Suresh et al. 2023).
- *Texture*: Grasshoppers have a crunchy exoskeleton, which may pose a challenge for some consumers unfamiliar with insect‐based foods (Haber et al. 2019).

### 12.1 Enhancing Taste and Texture Through Fermentation

Fermentation offers a transformative approach to improving the taste, texture, and overall appeal of grasshopper‐based products. By utilizing fermentation processes, the flavor profiles of grasshoppers can be enhanced, making them more palatable and appealing to a broader consumer base. Additionally, fermentation can improve the texture, helping to create products that mimic more traditional protein sources. While grasshoppers are nutritious, incorporating fermentation into their processing allows them to stand out as a more versatile and enjoyable ingredient, increasing their potential for market acceptance. This process can also improve digestibility and add unique flavors, which can help broaden their appeal.

- *Flavor optimization*: Beneficial microorganisms such as *Lactobacillus* are used to introduce savory, umami notes while reducing grassy or gamey flavors. This process enhances the palatability of grasshopper protein for mainstream consumers (Kewuyemi et al. 2020).
- *Texture modification*: Fermentation breaks down chitin, the main component of the grasshopper exoskeleton, softening it and creating a more pleasant mouthfeel suitable for diverse applications (Sang et al. 2020). This softening process makes the texture more pleasant, improving the mouthfeel and making it more suitable for various food applications. However, the breakdown of chitin might reduce its structural benefits, such as providing dietary fiber. However, some of the nutritional advantages of chitin can still be retained, depending on the fermentation process. The challenge is to balance texture improvement with preserving the health benefits of chitin, allowing for products that are both more enjoyable to eat and nutritionally beneficial.
- *Aromatic enhancements*: Fermentation can help neutralize any potential off‐putting odors that may be associated with grasshopper‐based products, which can be a concern for some consumers. While the smell might be more noticeable in raw or less processed forms, fermentation helps by breaking down compounds responsible for these odors, making the final product more appealing. Additionally, the fermentation process can introduce aromatic compounds that enhance the overall flavor profile, increasing product acceptance. This improvement in aroma and flavor balance may offset the initial concerns regarding odors, allowing grasshopper‐based products to appeal to a broader consumer base without detracting from their environmental and nutritional benefits (Haber et al. 2019; Cramer et al. 2017).
- *Nutritional enrichment*: Fermentation increases nutrient bioavailability and introduces probiotics, offering added health benefits such as improved digestion and gut health (Kewuyemi et al. 2020).

### 12.2 Applications for Fermented Grasshopper Products

Applications for fermented grasshopper products are as follows:

- *Fermented grasshopper condiments*: Grasshopper pastes and sauces can be fermented to produce savory condiments rich in umami flavors, similar to shrimp pastes, which are already used culinarily (Melton 2022).
- *Protein‐enriched foods*: Fermented grasshopper protein powders can be used in soups, smoothies, protein bars, and baked goods for added flavor and digestibility (Lisboa et al. 2024).
- *Texturized insect proteins (TIP)*: Combining fermentation with extrusion technology creates meat‐like textures, enabling grasshopper proteins to replace traditional meats in burgers, sausages, and patties.

### 12.3 Addressing Consumer Perception Through Innovation

Innovations in processing, such as fermentation, might help overcome consumer hesitancy. By mimicking familiar flavors and textures of traditional foods, grasshopper‐based products can appeal to a broader audience, including those new to entomophagy.

### 12.4 Integrating Halal Compliance and Sensory Appeal

When locust processing is combined with advanced processing techniques such as fermentation, their sensory qualities can be optimized. The integration of halal certification and sensory innovation should be beneficial for the acceptance of locust protein.

### 12.5 Traditional Culinary use in Muslim Countries

In many parts of the Middle East and Africa, locusts are considered a traditional delicacy. In Saudi Arabia, roasted locusts are consumed as snacks, especially during seasonal swarms. These are often seasoned with spices and served like roasted nuts. In rural parts of Pakistan and Yemen, locusts are fried or dried for storage and later consumption. The culinary familiarity in these regions has helped sustain entomophagy as part of cultural heritage (Khan et al. 2024; Suresh et al. 2023).

## 13 Future Perspectives on Halal Insect Protein in Mainstream Food Markets

As the edible insect industry continues to grow, there is significant potential for halal‐certified insect protein, including locust‐based products, to enter mainstream food markets. The following trends are likely to shape the future of halal insect protein:

- *Consumer acceptance*: Increasing consumer awareness of the environmental and nutritional benefits of insect protein, coupled with effective marketing and educational campaigns, can help overcome cultural resistance to insect‐based foods. Presenting locust‐based products in familiar formats, such as burgers or protein bars, will likely enhance consumer acceptance, particularly in Western markets (Cerritos 2009).

### 13.1 Regulatory and Certification Expansion

As the demand for halal‐certified products grows, regulatory frameworks and halal certification bodies will need to expand their guidelines to include insect‐based foods. This will ensure that locust‐based products meet halal standards, increasing consumer confidence and opening the door to new markets. Countries with established government‐sponsored halal certification processes, such as Malaysia and Indonesia, are likely to lead this regulatory expansion.

- *Innovation in food processing*: Advances in food processing technologies will enable producers to create more diverse and appealing grasshopper‐based products. Blending locust protein with other plant‐based ingredients or even with red meat and poultry can enhance the nutritional profile and make insect protein more palatable to a wider range of consumers. While mixing locust protein with red meat or poultry could add nutritional value and sustainability benefits, it requires careful formulation to balance flavor and texture. Innovations in packaging and branding can also help position locust‐based foods as both trendy and nutritious. Innovations in packaging and branding can also help position grasshopper‐based foods as both trendy and nutritious.
- *Processing and shelf life*: Insect‐based products, such as locust flour, are susceptible to lipid oxidation due to their high unsaturated fat content, leading to rancidity and diminished nutritional quality over time. De‐oiling techniques, like cold pressing, can mitigate this issue by reducing lipid content, thereby enhancing shelf life; however, these methods may slightly decrease protein levels (Wendin et al. 2020). Effective storage solutions are crucial for maintaining product quality. Vacuum‐sealed packaging, especially when combined with refrigeration at approximately 5°C, has been shown to significantly retard lipid oxidation and preserve sensory attributes over extended periods. Additionally, incorporating natural antioxidants, such as rosemary extract, into insect‐based products can further inhibit oxidative processes, thereby prolonging shelf life (Rossi et al. 2023). Pre‐treatment methods, including boiling, have demonstrated efficacy in reducing microbial loads in insect products. For instance, boiling house crickets for 1 min before refrigeration resulted in stable bacterial levels over more than 2 weeks of storage. Moreover, lactic acid fermentation of insect‐based composite flours has proven effective in controlling spoilage bacteria, contributing to extended shelf life and safety (Wendin et al. 2020). These findings underscore the importance of integrating appropriate processing techniques and storage conditions to ensure the safety, quality, and acceptability of insect‐based food products.

### 13.2 Global Market Integration

As the edible insect industry scales up, locust‐based products have the potential to move from niche markets to mainstream grocery stores and restaurants. Halal insect protein could become a staple in both Western and Muslim‐majority markets, addressing both sustainability concerns and consumer preferences for halal products.

## 14 Conclusions

The future of halal locust protein products is bright. With their halal status, environmental advantages, and nutritional benefits, locusts are well‐positioned to become a part of the global protein market. As consumer demand for sustainable and ethical food options continues to rise, locust‐based products have the potential to meet the needs of both halal‐conscious consumers and those seeking healthier, more sustainable alternatives to traditional meat. By addressing the challenges of certification, consumer education, and market penetration, the halal insect protein industry may have an important role in the future of food production. To enhance consumer acceptance in Muslim‐majority regions, further clarification on the halal status of different grasshopper species is necessary, supported by religious scholarship and scientific taxonomy.

Locust‐based food products represent a promising opportunity to address many of the world's most pressing food challenges, including sustainability, food security, and the rising demand for alternative proteins. With their high nutritional value, minimal environmental impact, and cultural and religious acceptance in Muslim‐majority regions, locust offers a viable solution as both a protein source and a sustainable alternative to conventional livestock, while grasshopper needs scholarly interpretation. As the global food system faces increasing pressure to reduce greenhouse gas emissions, land use, and water consumption, grasshoppers can significantly contribute to the development of a more resilient and eco‐friendly food supply.

Locusts, which are halal according to Islamic dietary laws, have the potential to fill an important gap in the market for alternative proteins that meet both ethical and religious standards, especially when the appropriate species are used. This broadens their appeal in regions like the United States, where kosher‐certified products are also in demand, and certain grasshopper species may qualify due to shared characteristics. Their high protein content, essential amino acids, healthy fats, and fiber provide a nutritionally dense option comparable to traditional animal proteins. Additionally, locust farming is highly efficient, requiring fewer resources and producing significantly lower greenhouse gas emissions, making it a sustainable choice for environmentally conscious consumers and regions with limited agricultural resources. Despite these advantages, the higher cost of locust‐based products can be attributed to limited production scale, processing complexity, and the initial investment required for insect farming systems. However, as production capacity increases and economies of scale are achieved, these costs are expected to decrease, improving accessibility and market competitiveness. Further research is needed to evaluate species‐specific halal rulings, establish standardized certification protocols for insect farming, and explore consumer acceptance strategies. In parallel, long‐term studies on allergenicity, nutritional bioavailability, and scalable farming models will support broader adoption of insect‐based proteins.

## Author Contributions

**Mian Nadeem Riaz**: conceptualization, resources, writing – review and editing, supervision. **Fariha Irshad**: writing – review and editing, methodology, investigation. **Awis Qurni Sazil**: writing – review and editing, resources.

## Conflicts of Interest

The authors declare no conflicts of interest.

## References

[crf370283-bib-0001] Abbasi, E. 2024. “A Review of Cultural Aspects and Barriers to the Consumption of Edible Insects.” Health Science Monitor 3, no. 3: 179–194. 10.61186/hsm.3.3.179.

[crf370283-bib-0002] Abbasi, E. , H. Alipour , M. Vahedi , and M. Mosashoar . 2021. “Halal Certification for Edible Insects.” Journal of Halal Research 4, no. 1: 58–67. 10.30502/h.2021.284875.1070.

[crf370283-bib-0003] Abdul‐Talib, A. N. , and I. S. Abd‐Razak . 2013. “Cultivating Export Market‐Oriented Behavior in Halal Marketing: Addressing the Issues and Challenges in Going Global.” Journal of Islamic Marketing 4, no. 2: 187–197.

[crf370283-bib-0004] Ademolu, K. O. , A. B. Idowu , and G. O. Olatunde . 2010. “Nutritional Value Assessment of Variegated Grasshopper, *Zonocerus Variegatus* (L.) (Acridoidea: Pygomorphidae), During Post‐Embryonic Development.” African Entomology 18, no. 2: 360–364. https://hdl.handle.net/10520/EJC32863.

[crf370283-bib-0005] Agbidye, F. S. , T. I. Ofuya , and S. O. Akindele . 2009. “Marketability and Nutritional Qualities of Some Edible Forest Insects in Benue State, Nigeria.” Pakistan Journal of Nutrition 8, no. 7: 917–922.

[crf370283-bib-0006] Akbar, J. , M. Gul , M. Jahangir , et al. 2023. “Global Trends in Halal Food Standards: A Review.” Foods 12, no. 23: 4200. 10.3390/foods12234200. 38231601 PMC10706363

[crf370283-bib-0007] Akhtar, Y. , and M. B. Isman . 2017. “Insects as an Alternative Protein Source.” In Proteins in Food Processing, 263–288. Elsevier.

[crf370283-bib-0008] Alexander, P. , C. Brown , A. Arneth , et al. 2017. “Could Consumption of Insects, Cultured Meat, or Imitation Meat Reduce Global Agricultural Land Use?” Global Food Security 15: 22–32.

[crf370283-bib-0009] Ali, B. , and J. M. Regenstein . 2014. “Halal Food Certification Challenges and Their Implications for Muslim Societies Worldwide.” International Periodical for the Languages, Literature and History of Turkish or Turkic 9: 111–130.

[crf370283-bib-0010] Ali, K. 2015. “Muslims and Meat‐Eating: Vegetarianism, Gender, and Identity.” Journal of Religious Ethics 43, no. 2: 268–288.

[crf370283-bib-0011] Al‐Qaradawi, Y. 1999. The Lawful and the Prohibited in Islam. Translated by K. El‐Helbawy M. M. Siddiqui , and S. Shukry . American Trust Publications.

[crf370283-bib-0012] Alzeer, J. , U. Rieder , and K. Abou Hadeed . 2018. “Rational and Practical Aspects of Halal and Tayyib in the Context of Food Safety.” Trends in Food Science & Technology 71: 264–267. 10.1016/j.tifs.2017.10.020.

[crf370283-bib-0013] Anjum, F. M. , M. S. Arshad , and S. Hussain . 2020. “Factory Farming and Halal Ethics.” In The Halal Food Handbook, John Wiley & Sons, 121–147. 10.1002/9781118823026.ch9.

[crf370283-bib-0014] Arshad, M. , G. Abbas , S. Jaffery , et al. 2022. “Towards Efficient Control of Locusts to Avoid Their Plagues on Humans: Evolving and Applying Advanced Control Strategies.” Pakistan Journal of Science 74, no. 5: 12–17.

[crf370283-bib-0015] Aydoğan, Z. , A. Taşer , Ü. İncekara , and A. Prastiwi . 2024. “Mineral Composition of Four Wild‐Harvested Edible Insects Consumed by the Indonesian People With an Updated List of Edible Insects in Indonesia.” *Research Square* .

[crf370283-bib-0016] Ayyub, R. M. 2015. “Exploring Perceptions of non‐Muslims Towards Halal Foods in UK.” British Food Journal 117, no. 9: 2328–2343. 10.1108/BFJ-07-2014-0257.

[crf370283-bib-0017] Azam, M. S. E. , and M. A. Abdullah . 2020. “Global Halal Industry: Realities and Opportunities.” International Journal of Islamic Business Ethics 5, no. 1: 47–59. 10.30659/ijibe.5.1.47-59.

[crf370283-bib-0018] Banjo, A. D. , O. A. Lawal , and E. A. Songonuga . 2006. “The Nutritional Value of Fourteen Species of Edible Insects in Southwestern Nigeria.” African Journal of Biotechnology 5, no. 3: 298–301. 10.5897/AJB05.250.

[crf370283-bib-0019] Bao, Y. , M. K. Leung , D. Poon , and C. Xiang . 2024. “Integrating Vertical Farm Into Low‐Carbon High‐Rise Building in High‐Density Context: A Design Case Study in Hong Kong.” Journal of Building Engineering 96: 110472.

[crf370283-bib-0020] Bhagat, S. , A. K. Santra , S. Mishra , et al. 2020. “The Water Footprint of Livestock Production System and Livestock Products: A Dark Area: A Review.” International Journal of Fauna and Biological Studies 7, no. 1: 83–88.

[crf370283-bib-0021] Blásquez, J. R. E. , J. M. P. Moreno , and V. H. M. Camacho . 2012. “Could Grasshoppers be a Nutritive Meal?” Food and Nutrition Sciences 3, no. 2: 164–175. 10.4236/fns.2012.32025.

[crf370283-bib-0022] Bonomini, M. G. , B. Prandi , and A. Caligiani . 2024. “Black Soldier Fly (*Hermetia illucens* L.) Whole and Fractionated Larvae: In Vitro Protein Digestibility and Effect of Lipid and Chitin Removal.” Food Research International 196: 115102. 10.1016/j.foodres.2024.115102. 39614512

[crf370283-bib-0023] Boukid, F. , G. Sogari , and C. M. Rosell . 2023. “Edible Insects as Foods: Mapping Scientific Publications and Product Launches in the Global Market (1996–2021).” Journal of Insects As Food and Feed 9, no. 3: 353–368.

[crf370283-bib-0024] Bukkens, S. G. 1997. “The Nutritional Value of Edible Insects.” Ecology of Food and Nutrition 36, no. 2–4: 287–319.

[crf370283-bib-0025] Cerritos, R. 2009. “Insects as Food: An Ecological, Social, and Economical Approach.” CABI Reviews: Perspectives in Agriculture, Veterinary Science, Nutrition and Natural Resources 4, no. 27: 1–10.

[crf370283-bib-0026] Cerritos, R. , and Z. Cano‐Santana . 2008. “Harvesting Grasshoppers *Sphenarium Purpurascens* in Mexico for Human Consumption: A Comparison With Insecticidal Control for Managing Pest Outbreaks.” Crop Protection 27, no. 3–5: 473–480. 10.1016/j.cropro.2007.08.001.

[crf370283-bib-0027] Cramer, H. , C. S. Kessler , T. Sundberg , et al. 2017. “Characteristics of Americans Choosing Vegetarian and Vegan Diets for Health Reasons.” Journal of Nutrition Education and Behavior 49, no. 7: 561–567. 10.1016/j.jneb.2017.04.011. 28689610

[crf370283-bib-0028] Das, M. , and S. Mandal . 2013. “Assessment of Nutritional Quality and Anti‐Nutrient Composition of two Edible Grasshoppers (Orthoptera: Acrididae)—A Search for new Food Alternative.” International Journal of Medicine and Pharmaceutical Science 3, no. 5: 31–48.

[crf370283-bib-0029] DeFoliart, G. R. 1991. “Insect Fatty Acids: Similar to Those of Poultry and Fish in Their Degree of Unsaturation, but Higher in the Polyunsaturates.” The Food Insects Newsletter 4, no. 1: 1–4.

[crf370283-bib-0030] Dossey, A. T. 2013. “Why Insects Should be in Your Diet.” Scientist 27, no. 2: 22–23.

[crf370283-bib-0031] Dossey, A. T. , J. T. Tatum , and W. L. McGill . 2016. “Modern Insect‐Based Food Industry: Current Status, Insect Processing Technology, and Recommendations Moving Forward.” In Insects as Sustainable Food Ingredients, 113–152. Academic Press.

[crf370283-bib-0032] Drewnowski, A. , M. J. Bruins , and J. J. Besselink . 2024. “Comparing Nutrient Profiles of Meat and Fish With Plant‐Based Alternatives: Analysis of Nutrients, Ingredients, and Fortification Patterns.” Nutrients 16, no. 16: 2725. 10.3390/nu16162725. 39203861 PMC11357199

[crf370283-bib-0033] Egonyu, J. P. , S. Subramanian , C. M. Tanga , T. Dubois , S. Ekesi , and S. Kelemu . 2021. “Global Overview of Locusts as Food, Feed and Other Uses.” Global Food Security 31: 100574.

[crf370283-bib-0034] El‐Mallakh, O. S. , and R. S. El‐Mallakh . 1994. “Insects of the Qur'an.” American Entomologist 40, no. 2: 82–84.

[crf370283-bib-0035] Gahukar, R. T. 2016. “Edible Insects Farming: Efficiency and Impact on Family Livelihood, Food Security, and Environment Compared With Livestock and Crops.” In Insects as Sustainable Food Ingredients, 85–111. Academic Press. 10.1016/B978-0-12-802856-8.00004-1.

[crf370283-bib-0036] Gotham, S. , and H. Song . 2013. “Non‐Swarming Grasshoppers Exhibit Density‐Dependent Phenotypic Plasticity Reminiscent of Swarming Locusts.” Journal of Insect Physiology 59, no. 11: 1151–1159. 10.1016/j.jinsphys.2013.08.017. 24035748

[crf370283-bib-0037] Govorushko, S. 2019. “Global Status of Insects as Food and Feed Source: A Review.” Trends in Food Science & Technology 91: 436–445.

[crf370283-bib-0038] Haber, M. , M. Mishyna , J. I. Martinez , and O. Benjamin . 2019. “The Influence of Grasshopper (*Schistocerca gregaria*) Powder Enrichment on Bread Nutritional and Sensorial Properties.” LWT 115: 108395.

[crf370283-bib-0039] Hassan, M. K. , M. M. Alshater , M. Rashid , and S. E. Hidayat . 2022. “Ten Years of the Journal of Islamic Marketing: A Bibliometric Analysis.” Journal of Islamic Marketing 13, no. 10: 2047–2068. 10.1108/JIMA-10-2020-0322.

[crf370283-bib-0041] Izberk‐Bilgin, E. , and C. C. Nakata . 2016. “A new Look at Faith‐Based Marketing: The Global Halal Market.” Business Horizons 59, no. 3: 285–292.

[crf370283-bib-0042] Jameel, S. 2023. “Climate Change, Food Systems and the Islamic Perspective on Alternative Proteins.” Trends in Food Science & Technology 138: 489–490. 10.1016/j.tifs.2023.06.028.

[crf370283-bib-0043] Kewuyemi, Y. O. , H. Kesa , C. E. Chinma , and O. A. Adebo . 2020. “Fermented Edible Insects for Promoting Food Security in Africa.” Insects 11, no. 5: 283. 10.3390/insects11050283. 32380684 PMC7290520

[crf370283-bib-0044] Khan, A. A. , A. A. Khan , and M. Meraj . 2024. “Exploring Locust Consumption Acceptance Factors Among Pakistani University Students: Insights for Sustainable Food Adoption.” Pakistan Journal of Humanities and Social Sciences 12, no. 2: 987–1000. 10.52131/pjhss.2024.v12i2.2158.

[crf370283-bib-0045] Khan, S. H. 2018. “Recent Advances in Role of Insects as Alternative Protein Source in Poultry Nutrition.” Journal of Applied Animal Research 46, no. 1: 1144–1157. 10.1080/09712119.2018.1474743.

[crf370283-bib-0046] Kim, S. Y. , S. Ji , J. H. Song , and S. Y. Kim . 2024. “Consumer Awareness and Attitudes Toward Insects.” Journal of Environmental Science International 33, no. 10: 765–772. 10.5322/jesi.2024.33.10.765.

[crf370283-bib-0047] Lever, J. , and M. Miele . 2012. “The Growth of Halal Meat Markets in Europe: An Exploration of the Supply Side Theory of Religion.” Journal of Rural Studies 28, no. 4: 528–537. 10.1016/j.jrurstud.2012.06.004.

[crf370283-bib-0048] Lipka, M. 2017. “Muslims and Islam: Key findings in the US and around the world.” https://coilink.org/20.500.12592/2bx9hc.

[crf370283-bib-0049] Lisboa, H. M. , A. Nascimento , A. Arruda , et al. 2024. “Unlocking the Potential of Insect‐Based Proteins: Sustainable Solutions for Global Food Security and Nutrition.” Foods 13, no. 12: 1846. 38928788 10.3390/foods13121846 PMC11203160

[crf370283-bib-0050] Majah, I. , and M. I. Yazid . 2015. “Sunan Ibn Majah.” In Studi Kitab Hadis. ‘Dari Muwaththa’ Imam Malik hingga Mustadrak al‐Hakim. Malang: Ahlimedia Press, 73–92.

[crf370283-bib-0051] Melton, L. 2022. “Noma: From Grasshopper Brews to Age‐old ‘Garum’.” Nature Biotechnology 40, no. 12: 1704. 10.1038/s41587-022-01622-6 36477506

[crf370283-bib-0052] Nirmal, N. , C. F. Anyimadu , A. C. Khanashyam , A. E. D. A. Bekhit , and B. K. Dhar . 2024. “Alternative Protein Sources: Addressing Global Food Security and Environmental Sustainability.” Sustainable Development 1–12.

[crf370283-bib-0053] Nizar, N. N. A. , S. A. S. Z. Abidin , A. Bujang , and M. N. Taib . 2022. “A Pre‐and Post‐Training Assessment of the Halal Executive Training Program Towards Upholding Halal Food Supply Chain.” Journal of Emerging Economies and Islamic Research 10, no. 2: 110–126.

[crf370283-bib-0054] Orsi, L. , L. L. Voege , and S. Stranieri . 2019. “Eating Edible Insects as Sustainable Food? Exploring the Determinants of Consumer Acceptance in Germany.” Food Research International 125: 108573. 31554134 10.1016/j.foodres.2019.108573

[crf370283-bib-0055] Palupi, E. , F. Uswah , I. Tanziha , et al. 2024. “Halal Status and Society Acceptance of Edible Insects.” Halal Studies and Society 1, no. 2: 24–29. 10.29244/hass.1.2.24-29.

[crf370283-bib-0056] Patel, S. 2019. “Insects as a Source of Sustainable Proteins.” In Proteins: Sustainable Source, Processing and Applications, 41–61. Academic Press (Imprint of Elsevier).

[crf370283-bib-0057] Priyatnasari, N. S. , E. Palupi , F. Kamila , K. R. Ardhiani , G. T. Prilyadi , and A. C. Iwansyah . 2024. “Meat‐Analog Made From Javanese Grasshopper, Kidney Beans, and Elephant Foot Yam as a High‐Protein and Low‐Cholesterol Product.” Journal of Agriculture and Food Research 16: 101071. 10.1016/j.jafr.2024.101071.

[crf370283-bib-0058] Raheem, D. , A. Raposo , O. B. Oluwole , M. Nieuwland , A. Saraiva , and C. Carrascosa . 2019. “Entomophagy: Nutritional, Ecological, Safety, and Legislation Aspects.” Food Research International 126: 108672. 31732082 10.1016/j.foodres.2019.108672

[crf370283-bib-0059] Regenstein, J. M. , M. M. Chaudry , and C. E. Regenstein . 2003. “The Kosher and Halal Food Laws.” Comprehensive Reviews in Food Science and Food Safety 2, no. 3: 111–127. 10.1111/j.1541-4337.2003.tb00018.x. 33451233

[crf370283-bib-0060] Riyaz, M. 2023. “Edible Insects: Islamic Perspectives on Entomophagy and Future Foods: Islamic Perspectives on Edible Insects.” SHAHIH: Journal of Islamicate Multidisciplinary 8, no. 2: 63–80. 10.22515/shahih.v8i2.7670.

[crf370283-bib-0061] Rossi, G. , S. Ojha , N. Pathak , P. Mahajan , and O. K. Schlüter . 2023. “Storage and Packaging of Edible Insects.” In Edible Insects Processing for Food and Feed, 261–276. CRC Press. 10.5539/jfr.v7n1p21.

[crf370283-bib-0062] Rumpold, B. A. , and O. K. Schlüter . 2013. “Nutritional Composition and Safety Aspects of Edible Insects.” Molecular Nutrition & Food Research 57, no. 5: 802–823. 23471778 10.1002/mnfr.201200735

[crf370283-bib-0063] Sabri, N. S. A. , M. I. F. Kamardan , S. X. Wong , et al. 2023. “Future Aspects of Insects' Ingestion in Malaysia and Indonesia for Human Well‐Being and Religion Regulation.” Future Foods 8: 100267. 10.1016/j.fufo.2023.100267.

[crf370283-bib-0064] Sang, X. , X. Ma , H. Hao , J. Bi , G. Zhang , and H. Hou . 2020. “Evaluation of Biogenic Amines and Microbial Composition in the Chinese Traditional Fermented Food Grasshopper Sub Shrimp Paste.” LWT 134: 109979.

[crf370283-bib-0065] Sani, N. A. , and H. A. Dahlan . 2015. “Current Trend for Food Safety and Halal Measures.” Paper presented at the ASEAN Community Conference, Bangi, Malaysia, November 11–12.

[crf370283-bib-0066] Šerifović, J. , A. Dugonjić , S. Šušnić , N. Uršulin‐Trstenjak , and S. Haliti . 2022. “Sustainable Halal Food Production—Locusta Migratoria as Unused Potential.” Ekonomski Izazovi 11, no. 22: 74–80.

[crf370283-bib-0067] Shamsuddin, M. M. J. 2024. “Entomophagy and Islamic Jurisprudence: Examining the Concept of “Al‐Khabīth” in Contemporary Dietary Discourse.” Al‐Qanatir: International Journal of Islamic Studies 33, no. 6: 212–230.

[crf370283-bib-0068] Sharawi, S. E. 2024. “Exploring the Various Orders of Edible Insects in the Kingdom of Saudi Arabia as a Safe and Sustainable Food Alternative: A Comprehensive Review.” Journal of Applied & Natural Science 16, no. 4: 1393–1401.

[crf370283-bib-0069] Shrivastava, S. K. , A. Prakash , J. Rao , S. Saxena , and M. Arif . 2019. “Grasshopper: An Untapped Halal Food Source for Future India.” Journal of Applied Zoological Research 30, no. 2: 93–132.

[crf370283-bib-0070] Siddiqui, A. H. 1976. Sahih Muslim (English trans.). Kazi Publications (Peace Vision). ISBN 978‐0686183419.

[crf370283-bib-0071] Siddiqui, S. A. , M. Ghisletta , B. M. Yunusa , et al. 2023. “Grasshoppers and Locusts as human Foods—A Comprehensive Review.” Journal of Insects As Food and Feed 9, no. 10: 1247–1264.

[crf370283-bib-0072] Song, H. 2010. “Grasshopper Systematics: Past, Present and Future.” Journal of Orthoptera Research 19, no. 1: 57–68. https://www.jstor.org/stable/20789567.

[crf370283-bib-0073] Sultana, R. , S. Kumar , A. A. Samejo , S. Soomro , and M. Lecoq . 2021. “The 2019–2020 Upsurge of the Desert Locust and its Impact in Pakistan.” Journal of Orthoptera Research 30, no. 2: 145–154. 10.3897/jor.30.65971.

[crf370283-bib-1080] Sunan Ibn Majah . 2007. Sunan Ibn Majah. Book 27, Hadith 3238. Translated by N. al‐Khattab. Darussalam.

[crf370283-bib-0074] Suresh, S. , N. S. M. Zaini , M. H. Abd Rahim , and N. H. Ahmad . 2023. “Insects and Worms as an Alternative Protein Source in the Halal Food Industry.” In Innovation of Food Products in Halal Supply Chain Worldwide, 127–148. Academic Press. 10.1016/B978-0-323-91662-2.00012-0.

[crf370283-bib-0075] Tajudeen, A. L. 2020. “Halal Certification of Insect‐Based Food: A Critique.” IJIBE (International Journal of Islamic Business Ethics) 5, no. 2: 100–112. 10.30659/ijibe.5.2.100-112.

[crf370283-bib-0076] Van Broekhoven, S. , D. G. Oonincx , A. Van Huis , and J. J. Van Loon . 2015. “Growth Performance and Feed Conversion Efficiency of Three Edible Mealworm Species (Coleoptera: Tenebrionidae) on Diets Composed of Organic By‐Products.” Journal of Insect Physiology 73: 1–10. 10.1016/j.jinsphys.2014.12.005. 25576652

[crf370283-bib-0077] Van Huis, A. 2013. “Potential of Insects as Food and Feed in Assuring Food Security.” Annual Review of Entomology 58, no. 1: 563–583. 10.1146/annurev-ento-120811-153704. 23020616

[crf370283-bib-0078] Van Huis, A. , and D. G. Oonincx . 2017. “The Environmental Sustainability of Insects as Food and Feed: A Review.” Agronomy for Sustainable Development 37: 1–14. 10.1007/s13593-017-0452-8.

[crf370283-bib-0079] Wendin, K. , L. Mårtensson , H. Djerf , and M. Langton . 2020. “Product Quality During the Storage of Foods With Insects as an Ingredient: Impact of Particle Size, Antioxidant, Oil Content and Salt Content.” Foods 9, no. 6: 791. 10.3390/foods9060791. 32560134 PMC7353477

[crf370283-bib-0080] WHO . 2007. Protein and amino acid requirements in human nutrition . World Health Organization Technical Report Series 935. WHO. 18330140

[crf370283-bib-0081] Wilkins, S. , M. M. Butt , F. Shams , and A. Pérez . 2019. “The Acceptance of Halal Food in non‐Muslim Countries: Effects of Religious Identity, National Identification, Consumer Ethnocentrism and Consumer Cosmopolitanism.” Journal of Islamic Marketing 10, no. 4: 1308–1331. 10.1108/JIMA-11-2017-0132.

[crf370283-bib-0082] Wu, G. , J. Fanzo , D. D. Miller , et al. 2014. “Production and Supply of High‐quality Food Protein for human Consumption: Sustainability, Challenges, and Innovations.” Annals of the New York Academy of Sciences 1321, no. 1: 1–19. 10.1111/nyas.12500. 25123207

[crf370283-bib-0083] Yi, L. , C. M. Lakemond , L. M. Sagis , V. Eisner‐Schadler , A. Van Huis , and M. A. van Boekel . 2013. “Extraction and Characterisation of Protein Fractions From Five Insect Species.” Food Chemistry 141, no. 4: 3341–3348. 10.1016/j.foodchem.2013.05.115. 23993491

